# Impact of early percutaneous dilatative tracheostomy in patients with subarachnoid hemorrhage on main cerebral, hemodynamic, and respiratory variables: A prospective observational study

**DOI:** 10.3389/fneur.2023.1105568

**Published:** 2023-03-27

**Authors:** Giovanni Bini, Emanuele Russo, Marta Velia Antonini, Erika Pirini, Valentina Brunelli, Fabrizio Zumbo, Giorgia Pronti, Alice Rasi, Vanni Agnoletti

**Affiliations:** ^1^Department of Emergency Surgery and Trauma, Anesthesia and Intensive Care Unit, M Bufalini Hospital, Azienda Unità Sanitaria Locale (AUSL) della Romagna, Cesena, Italy; ^2^Department of Biomedical, Metabolic and Neural Sciences, University of Modena and Reggio Emilia, Modena, Emilia-Romagna, Italy; ^3^Neurointensive Care Unit, ASST Grande Ospedale Metropolitano Niguarda, Milan, Italy; ^4^Department of Anesthesia and Intensive Care, Osspedale degli Infermi, Rimini, Italy; ^5^Department of Pediatrics, Ospedale Bufalini, Cesena (FC), Italy

**Keywords:** neurocritical care, intracranial pressure, subarachanoid hemorrhage, percutaneous tracheostomy, critical care

## Abstract

**Introduction:**

Patients with poor-grade subarachnoid hemorrhage (SAH) admitted to the intensive care unit (ICU) often require prolonged invasive mechanical ventilation due to prolonged time to obtain neurological recovery. Impairment of consciousness and airway protective mechanisms usually require tracheostomy during the ICU stay to facilitate weaning from sedation, promote neurological assessment, and reduce mechanical ventilation (MV) duration and associated complications. Percutaneous dilatational tracheostomy (PDT) is the technique of choice for performing a tracheostomy. However, it could be associated with particular risks in neurocritical care patients, potentially increasing the risk of secondary brain damage.

**Methods:**

We conducted a single-center, prospective, observational study aimed to assess PDT-associated variations in main cerebral, hemodynamic, and respiratory variables, the occurrence of tracheostomy-related complications, and their relationship with outcomes in adult patients with SAH admitted to the ICU of a neurosurgery/neurocritical care hub center after aneurysm control through clipping or coiling and undergoing early PDT.

**Results:**

We observed a temporary increase in ICP during early PDT; this increase was statistically significant in patients presenting with higher therapy intensity level (TIL) at the time of the procedural. The episodes of intracranial hypertension were brief, and appeared mainly due to the activation of cerebral autoregulatory mechanisms in patients with impaired compensatory mechanisms and compliance.

**Discussion:**

The low number of observed complications might be related to our organizational strategy, all based on a dedicated “tracheo-team” implementing both PDT following a strictly defined protocol and accurate follow-up.

## Highlights

- To our knowledge, this is the first prospective study performed exclusively on patients suffering from poor-grade SAH to evaluate ICP variations during PDT.
- PDT is not without risk, but intra-procedure intracranial hypertension and CPP reduction are transient, and do not impact clinical outcomes.
- Before performing a tracheostomy, current TIL and ICP variations during EDV clamping test should be assessed; if there is concern about intracranial hypertension and or EDV clamp intolerance, the procedure should be deferred.
- A specialized tracheo-team allows standardization of the procedure and treatment of any complications through dedicated protocols, thus minimizing the risks.
- In patients with acute brain injury the recommended timing for PDT is not defined; in poor-grade SAH it is meaningful to consider PDT after early brain injury and before the second hit of vasospasm (day III–V).

## Introduction

Subarachnoid hemorrhage (SAH) is a severe clinical entity that mainly affects patients between 55 and 60 years of age, primarily affecting females (60–70% of cases). SAH has been associated with a variable mortality rate of 30–50% and a favorable outcome ranging from 25 to 58% (1–6).

Patients with poor-grade subarachnoid hemorrhage (1, 7), as per the World Federation of Neurosurgical Societies (WFNS) score (8) and Hunt and Hess (HH) scale (9), admitted to the intensive care unit (ICU) often require prolonged hospitalization. Slow recovery of consciousness and impaired airway protective mechanisms complicate extubation, eventually impacting the reliability of conventional predictor criteria for successful extubation (10–12). When the need for invasive mechanical ventilation (MV) beyond 14 days is expected, performing tracheostomy, rather than prolonged tracheal intubation, might be advisable (13, 14).

In the recent consensus for mechanical ventilation in patients with acute brain injury released by the European Society of Intensive Care Medicine (ESICM) (15), no recommendation on optimal timing for tracheostomy was provided due to contradictory low quality evidence available at the time of writing. The limited evidence supporting early tracheostomy in neurocritical patients, (6, 16–23) and, specifically in SAH (5, 24), suggests an increased patient comfort, potential facilitation of sedation weaning, an improvement in the feasibility of neurological assessment, and potentially, a decrease in the duration of MV and the risk of ventilator-associated events (VAEs), and a lower ICU length of stay (LOS) and the associated costs. However, to date, no definitive recommendations exist supporting the standardization of timing for tracheostomy in this population (15).

Tracheostomy is a well-established procedure commonly performed bedside in the ICU. A broad consensus supports the choice of percutaneous dilatational tracheostomy (PDT) as the preferred method compared with surgical tracheostomy (25–31). In patients with brain injury, PDT could be associated with peculiar risks, such as hypoxia, hypercapnia, temporary apnea, systemic arterial hypertension, and hyperextension of the neck, which can induce intracranial hypertension (HICP), defined as ICP above 20 mm Hg (1, 3, 4, 13, 32, 33), thus increasing the risk of secondary brain damage.

To date, there are no conclusive findings or recommendations on the indications for ICP monitoring in patients suffering from SAH. In the case of acute symptomatic hydrocephalus following a SAH, the American Heart Association and American Stroke Association recommendations suggested inserting an intraventricular catheter (34). In patients suffering from SAH, the % of ICP monitoring is variable among different centers; Baggiani et al. (35) reports 69% ICP monitored patients (inter-center variability from 6.4 to 82.1%), and out of them, 54.9% had external ventricular catheter; in poor grades (WFNS IV–V), the percentage is 73%. Intracranial hypertension is recorded in 54.7% of cases; in patients with DVE, the incidence of ICP > 20 mmHg is lower (46 vs. 75%). ICP monitoring appears to be associated with lower rates of unfavorable outcomes (35).

Many authors have investigated the variability in intracranial pressure (ICP) during tracheostomy in heterogeneous populations of neurocritical care patients with traumatic brain injury (TBI), intracranial hemorrhage (ICH), ischemic stroke, brain infections, and neoplastic disease. All of these present with variable pathophysiology, clinical course, and outcomes (13, 14, 32–38).

In this single-center prospective analysis, we aimed to assess the incidence of HICP and impaired hemodynamic and respiratory variables during early PDT in a cohort of patients exclusively with subarachnoid hemorrhage.

## Methods

We conducted a single-center, prospective, observational study that included all consecutive adult patients with SAH admitted to the Intensive Care Unit of the Maurizio Bufalini Hospital, AUSL della Romagna (Azienda-Unità Sanitaria Locale, Local Healthcare Authority), Cesena, Italy (hub hospital for neurosurgery and neurocritical intensive care) from August 2017 to December 2020. The Ethical Committee of the AUSL della Romagna (Comitato Etico della Romagna, C.E.Rom.) reviewed and approved the study protocol (determination n° 2290), which met the Helsinki Declaration guidelines (39).

Inclusion criteria were age above 18 years, SAH admission diagnosis, aneurysm treatment with coiling or clipping, the requirement for PDT, and ICP monitoring during PDT. Patients without surgical or endovascular intervention due to ultra-early poor prognosis, no need for PDT, and a lack of ICP monitoring during PDT were excluded from the study.

A prognosis assessment, performed on the 2nd or 3rd day after ICU admission, identified the likelihood of prolonged ventilatory support (>14 days); the attending neurointensivists were in charge of the final decision to perform a tracheostomy. The presence of coagulation disorders, previous tracheostomies, goiter, anatomical abnormalities dislocating the trachea, or involving peritracheal blood vessels were considered absolute contraindications to PDT, and the presence of sustained ICP increases >20 mm Hg for at least 5 minutes during the previous 24 h, leading to a change in therapy intensity level (TIL) scale, were considered relative contraindications to PDT.

The primary outcome was the incidence of HICP, defined as ICP above 20 mmHg, during early PDT. The secondary endpoints were the incidence of periprocedural low cerebral perfusion pressure (CPP), below 65 mmHg, and hypercapnia, PaCO_2_, above 45 mm Hg, the frequency of early tracheostomy (performed in the first 7 days after an event) and late tracheostomy (performed after 7 days following an event) complications, neurological outcomes at 6 months, and mortality.

We recorded injury severity at ICU admission according to the WFNS score (8), HH scale (9), and Fisher scale (40) based on the first brain computed tomography, if an endovascular or surgical approach was used, and if decompressive craniectomy was performed during the first days after admission, before PDT. Days on invasive mechanical ventilation before PDT, the intensity of interventions according to TIL score (41), analgesia and sedation strategy, and other additional data were also collected (Table 1).

**Table 1:** Baseline patients’ characteristics, neurological injury severity, and associated interventions in the overall population of adult patients with SAH adult admitted during the study period after aneurysm control through clipping or coiling (*n* = 50).

			Total *n* = 50	Normal ICP *n* = 30	High ICP *n* = 20	*p*-value*
**Baseline characteristics**						
Age (years)		Mean (SD)	59 (±12)	60 (±13)	58 (±10)	0.523
Sex (female)		*n* (%)	34 (68)	22 (73)	12 (60)	0.322
**Severity grading scores**						
WFNS score	1	*n* (%)	0 (0)	0 (0)	0 (0)	0.403
	2	*n* (%)	4 (8)	2 (7)	2 (7)	
	3	*n* (%)	8 (16)	4 (13)	4 (20)	
	4	*n* (%)	17 (34)	13 (43)	4 (20)	
	5	*n* (%)	21 (42)	11 (37)	10 (50)	
HH scale	1	*n* (%)	0 (0)	0 (0)	0 (0)	0.631
	2	*n* (%)	4 (8)	2 (7)	2 (10)	
	3	*n* (%)	8 (16)	4 (13)	4 (20)	
	4	*n* (%)	15 (30)	11 (37)	4 (20)	
	5	*n* (%)	23 (46)	13 (43)	10 (50)	
Fisher scale	1	*n* (%)	0 (0)	0 (0)	0 (0)	0.195
	2	*n* (%)	2 (4)	0 (0)	2 (10)	
	3	*n* (%)	17 (34)	10 (33)	7 (35)	
	4	*n* (%)	31 (62)	20 (67)	11 (55)	
TIL scale	1	*n* (%)	6 (12)	3 (10)	3 (15)	0.005
	2	*n* (%)	21 (42)	18 (60)	3 (15)	
	3	*n* (%)	19 (38)	6 (20)	13 (65)	
	4	*n* (%)	4 (8)	3 (10)	1 (5)	
**Interventions**						
ICP catheter, ventricular		*n* (%)	46 (92)	28 (93)	18 (90)	0.670
ICP catheter, parenchymal		*n* (%)	4 (8)	2 (7)	2 (10)	
Pre-PDT coiling		*n* (%)	22 (44)	11 (37)	11 (55)	0.201
Pre-PDT clipping		*n* (%)	28 (56)	19 (63)	9 (45)	
Pre-PDT decompressive craniectomy		*n* (%)	6 (12)	5 (16)	1 (5)	0.214

ICP, intracranial pressure; SD, standard deviation; WFNS, World Federation of Neurosurgical Societies; HH, hunt and hess; TIL, therapy intensity level scale; PDT, percutaneous dilatational tracheostomy. The group is also stratified according to normal (*n* = 30) versus high ICP status (*n* = 20).

TIL score ranges from 0 (no ICP-directed therapy) to 4 (extreme therapy). TIL takes into account the following items: osmotic therapy, ventilation and PaCO_2_ targets, temperature targets, sedation levels, vasopressors infusion, CSF drainage, and decompressive craniectomy (41).

### Variables and measurement

Procedural vital signs (ICP, CPP, mean arterial pressure (MAP), heart rate (HR), SpO_2_, EtCO_2_ - (Drager Infinity C700 for IT monitor, Drager Medical AG & Co KG D-23542 Lubeck Germany), as PaO2 and PaCO2 (RAPID Systems blood gas analyzer, Siemens Healthcare Diagnostics Inc. 511 Benedict Avenue Tarrytown NY 10591-5097, United States), were recorded at five predetermined timepoints: T1, baseline, T2, patient positioning, T3, skin incision, T4, cannulation, and T5, 10 min after completion of the procedure (32, 33, 36, 37) (Tables 2, 3). The duration of the procedure, from skin incision until EtCO2 waveform recovery, (14, 42) has been reported. The hourly cerebrospinal fluid (CSF) drainage volume in the 24 h prior to the procedure was recorded.

**Table 2:** Baseline tracheostomy and pre-tracheostomy data in the overall population and stratified according to normal versus high ICP status.

		Total *n* = 50	Normal ICP *n* = 30	High ICP *n* = 20	*p*-value*
**Tracheostomy**					
Day of procedure	Mean (SD)	4.3 (±2.1)	4.4 (±1.7)	4.4 (±2.7)	0.445
	Median (IQR)	4 (3)	4 (3)	4 (3)	
Early tracheostomy (≤7 days)	*n* (%)	47 (94)	29 (96)	18 (90)	0.324
Tracheostomy duration (minutes)	Mean (SD)	8.9 (±7.7)*	8 (±6)	10 (±8)	0.418
**Pre-tracheostomy data**					
ICP (mm Hg)	Mean (SD)	12 (±5)	10 (±5)	15 (±3)	0.442
FiO_2_	Mean (SD)	0.45 (±0.7)	44.7 (±6.3)	46.5 (±8.8)	0.425
PEEP (mm Hg)	Mean (SD)	6.8 (±1.9)	6.7 (±1.9)	6.7 (±1.9)	0.442
BT^§^ (°C)	Mean (SD)	37.4 (±0,7)	37.4	37.4	0.921
Na^+^ plasma concentrations (mmol/L)	Mean (SD)	148	149	147	0.233

ICP, intracranial pressure; SD, standard deviation; IQR, interquartile range; FiO_2_, fraction of inspired oxygen; PEEP, positive end-expiratory pressure; BT, body temperature; Na^+^, sodium; aPTT, activated partial thromboplastin time; INR, international normalized ratio; PLT, platelets.

**Table 3:** Variation of main parameters recorded at five predetermined time points during the tracheostomy procedure.

Timepoint	Baseline T1	Positioning T2	Incision T3	Cannulation T4	End T5	*p* ANOVA test
ICP (mm Hg)	12.6 (±5.6)	14.6 (±5.8)	17.1 (±7.1)	19.1 (±8.5)	11.4 (±5.5)	<0.0001
MAP (mm Hg)	87.1 (±16.5)	86.4 (±16.7)	90.2 (±14)	95.1 (±17.4)	86.4 (±12.5)	0.020
CPP (mm Hg)	86.4 (±12.5)	70.8 (±13.8)	72 (±14.6)	74.9 (±15)	75.1 (±10.9)	
PaO_2_ (mm Hg)	166 (±111)	351 (±121)	359 (±10)	366 (±115)	189 (±139)	<0.0001
PaCO_2_ (mm Hg)	39 (±5)	37 (±5)	38 (±5)	39 (±1)	35 (±5)	

ICP, intracranial pressure; MAP, mean arterial pressure;CPP, cerebral perfusion pressure; PaO2, arterial partial pressure of oxygen; PaCO2, arterial partial pressure of carbon dioxide.

Patients with ICP values >20 mmHg (32, 33, 36, 37) requiring additional therapies for the treatment of raised ICP were classified as high-ICP (H-ICP); patients who did not present with ICP values >20 mmHg were defined as having normal ICP (N-ICP). The threshold for low CPP was set at 65 mmHg in this population (1).

### Procedure

Tracheostomy was performed by a team of experienced physicians and nurses, consistently with a previously published protocol (43), according to the Ciaglia approach (44, 45), and using the “Blue Rhino” set (Cook Critical Care, Bloomington, IN). Continuous airway monitoring through bronchoscopy was performed during the procedure (46, 47) over an endotracheal tube (7.5/8 mm OD) (48). A moderate anti-Trendelenburg position (20°) with neck hyperextension was obtained, guided by ICP monitoring, and maintained throughout PDT. The patients were managed with total intravenous anesthesia (midazolam or propofol combined with sufentanil and remifentanil) and muscular paralysis (rocuronium bromide or cisatracurium besylate). They were under controlled ventilation (intermittent positive pressure ventilation, IPPV), with a tidal volume (Vt) of 6–8 ml/kg, respiratory rate (RR) titrated to PaCO2, and a fraction of inspired oxygen (FiO_2_) of 1.

Prior to the procedure, the EVD is held open (49, 50) with hourly clamping to measure ICP. The initial EVD setting was +10 cmH2O. At the skin incision point, the EVD was clamped, allowing ICP monitoring during PDT.

In an episode of ICP elevation above 20 mmHg for at least 1 minute (1, 4), a management protocol was performed: a single shot of 5 ml liquor drainage (if ventricular catheter in place), then any or more of the following interventions, on a case by case basis: temporary removal of the fiber-optic endoscope to improve mechanical ventilation, or hyperventilation (if EtCO_2_ increased >5 mm Hg with a fiber-optic endoscope inserted), a bolus of hypertonic saline solution (NaCl 3%, 250 ml) or mannitol 18% (0.5 mg/kg), a bolus of sedation, and increasing vasopressor (norepinephrine) dosage (1, 4, 32, 36, 51).

### Outcome

Periprocedural complications have been reported, particularly accidental extubation, posterior tracheal wall puncture, tracheal ring rupture, false passage, pneumothorax, and bleeding (self-limiting or requiring treatment). Any interruption in the procedure to manage eventual complications or the requirement for transition to surgical tracheostomy was recorded.

The time on post-PDT mechanical ventilation was defined as the number of days from tracheostomy to full weaning from MV, ICU LOS was defined as the number of days from ICU admission to discharge, hospital LOS as the number of days from hospital admission until discharge, and cannula time as the number of days from tracheostomy until final decannulation.

A team of experienced neurointensive care nurses performed telephone interviews to assess complications (range 3–6 months) such as local infection (signs of inflammation/infections and cellulitis), late bleeding requiring interventions, granulomas, or tracheal stenosis requiring specific treatment. The Glasgow Outcome Scale (GOS) was used to assess neurological outcomes (52).

### Study size

All patients meeting inclusion criteria and for whom no data were missed during the study period were enrolled.

### Statistical analysis

Demographic and clinical data were reported using descriptive statistics. Quantitative variables were reported as median values (interquartile range, IQR) or mean (standard deviation, SD), while qualitative variables were reported as numbers (absolute value and %). Independent Student’s *t*-test, Mann–Whitney U test, and *χ*^2^ tests were used for statistical analysis, performed using IBM SPSS 22.0.

### Reporting

The STROBE statement for reporting observational studies in epidemiology was followed (53).

## Results

A total of 297 patients with SAH were admitted to the intensive care unit of Bufalini Hospital, AUSL della Romagna, Italy, from August 1, 2017, to December 31, 2020. Sixty patients did not undergo surgical or endovascular aneurysm treatment because of expected futility. Twenty-four of these experienced early death, according to neurological determination (DND). Thirty-six underwent early withdrawal of life-sustaining treatment (WLST) after extensive neuro-prognostication, performed 72 h following the initial event, consistently with current recommendations (54–56). The decision was shared with the families.

A total of 237 patients with SAH underwent coiling or clipping, 85 of whom underwent PDT. In 23 patients, ICP monitoring was not performed during tracheostomy and was excluded from the main analysis. Data were not completely available for 12 procedures because of organizational issues.

**Figure 1:**
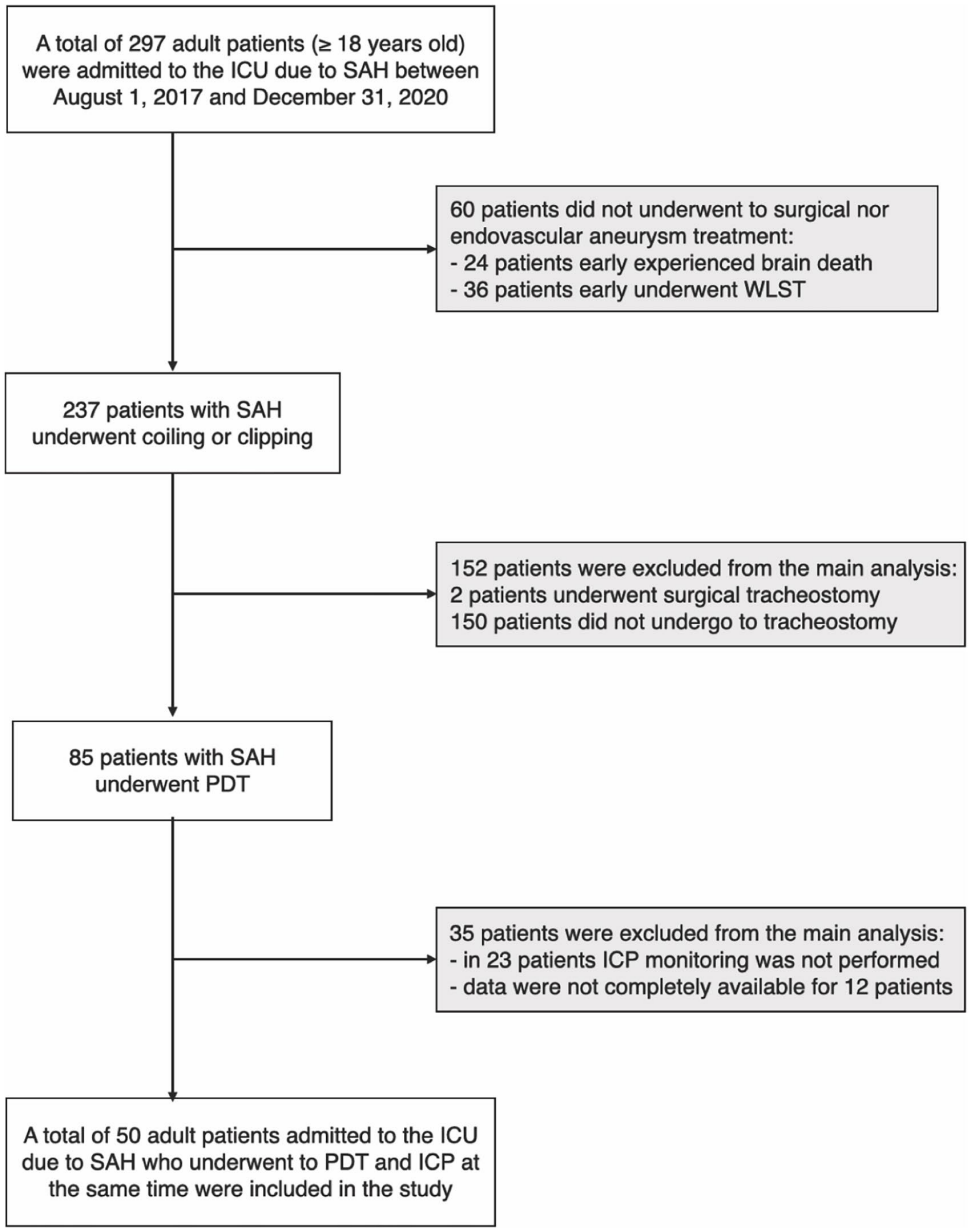
Flow chart of study population selection; in the gray boxes, the subjects were excluded from the main analysis (see methods). ICU, intensive care unit; SAH, subarachnoid hemorrhage; WLST, withdrawal of life-sustaining treatments; PDT, percutaneous dilational tracheostomy; ICP, intracranial pressure.

Fifty adult patients were included in the main analysis (Figure 1); characteristics of study participants are included in Table 1. Intracranial pressure was monitored through an external ventricular drain (EVD) in 46 patients (92%) and an intraparenchymal probe in four patients (8%). Hourly output of CSF in 24 h pre tracheostomy was 9.3 ± 2.6 (SD) ml/h in 46 patients with EVD.

The mean age of the patients was 59 years (SD ± 12 years), and 34 (68%) were female. Thirty-eight patients (76%) presented with poor-grade SAH at ICU admission (WFNS and HH scores IV–V). On the first brain CT scan, 48 patients (96%) scored 3–4 on the Fisher scale.

Obliteration of the aneurysm occurred through surgical clipping in 28 patients (56%) and through coiling in the remaining 22 patients (44%). Decompressive craniectomy was performed in six patients (12%). SAH severity grading scores, and associated interventions are shown in Table 1. PDT was performed after a median of 4 days (IQR 3; mean 4.3, ±2.1). In 46 patients (94%), a tracheostomy was performed within the first week after ICU admission (Table 2).

### Primary endpoint

The ICP value increased significantly from 12.6 mmHg (±5.6) at baseline (T1) to 17.1 mm Hg (±7.1) at skin incision (T3) to 19.1 mm Hg (±8.5) at cannulation (T4), then decreased to 11.4 mm Hg (±5.5) at the end of the procedure (Table 3).

An episode of ICP > 20 mmHg occurred in 27 patients (54%) in at least one of the five time points. In seven of them (26% of the raised ICP group), according to the limited duration and severity of the raised ICP episodes, no treatment was deemed necessary; in the remaining 20 (74%), interventions aimed at lowering ICP were implemented according to the previously described protocol. These 20 patients were included in the H-ICP group. The most commonly performed interventions were cerebrospinal fluid withdrawal (30% of patients in the H-ICP group), endoscope removal and ventilation or temporary hyperventilation (24%), additional sedation bolus (14%), and osmotic therapy (10%).

In H-ICP hourly output of CSF in the previous 24 h was 9.5 ± 2.5 ml/h vs. 9.2 ± 2.5 ml/h in N-ICP; in 15 patients with CSF withdrawal during PDT, hourly 24 h CSF output was 9.5 ± 2.4 ml/h, vs. 9.3 ± 2.5 in patients without CSF drainage.

**Figure 2:**
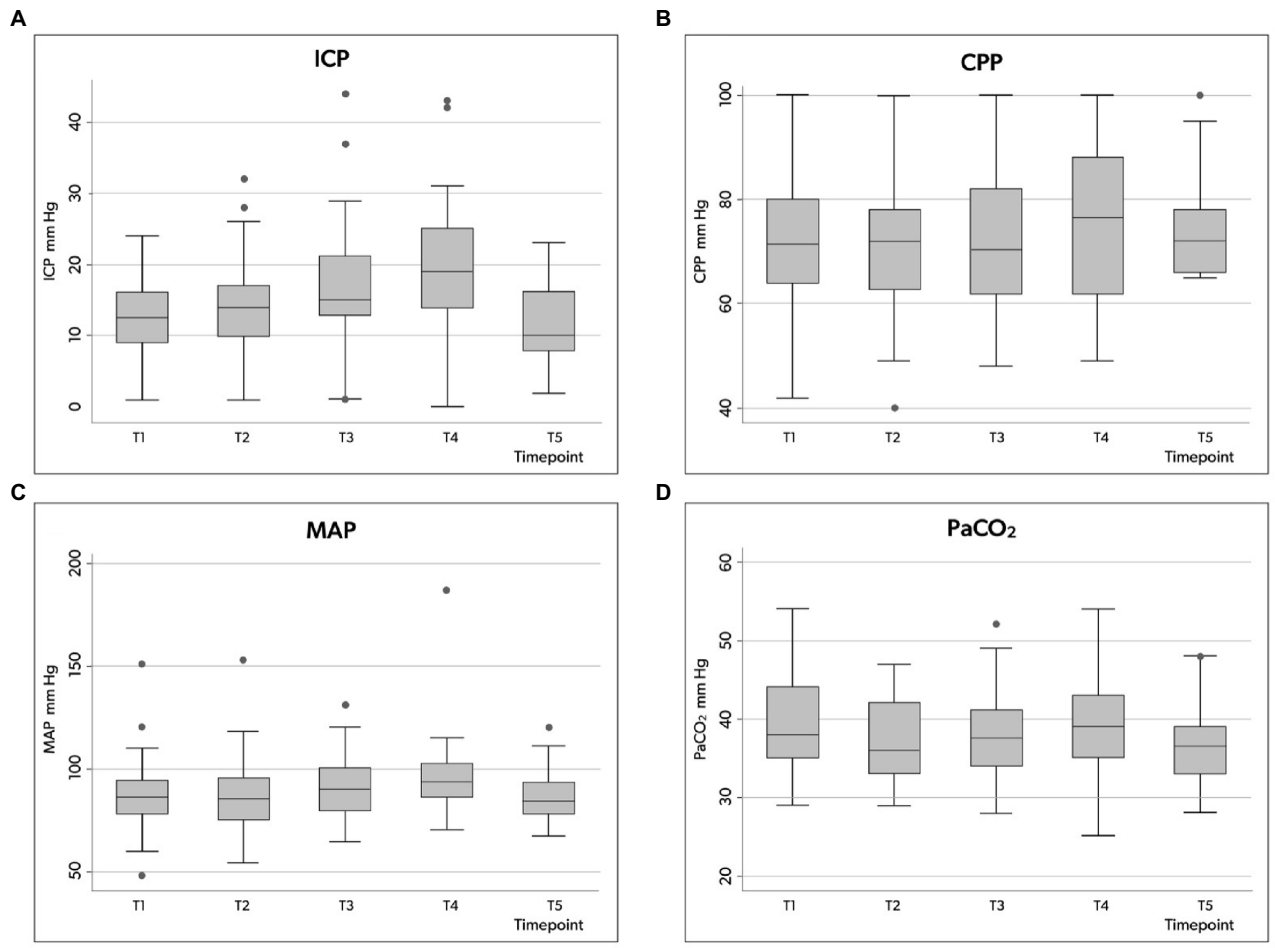
Box plots illustrating the distribution of ICP **(A)**, CPP **(B)**, MAP **(C)** and PaCO2 **(D)** values (minimum value, first quartile, median, third quartile, and maximum value) at the five time points in the whole group. ICP, intracranial pressure; CPP, cerebral perfusion pressure; MAP, mean arterial pressure; PaCO_2_, partial pressure of carbon dioxide in the arterial blood.

Compared to the N-ICP group, H-ICP group presented significantly higher ICP values at T1, T4, and T5 (Figure 2A). Particularly, in the H-ICP group, a mean ICP of 22.3 mm Hg (±7.3) and 25.7 mm Hg (±8.0) at incision and cannulation, respectively. At T4, in 17 patients (34%), ICP increased above 20 mm Hg, and in 12 (24%), values increased to >25 mmHg. In these subgroups, at T5, the mean ICP values decreased to 13 mm Hg (±5) and 14 mm Hg (±5) mmHg, respectively. ICP decreased to 14.0 mm Hg (±5.1) at the end of the procedure (Figure 2A). In only one patient, an ICP value above 20 mmHg was recorded at T5; ICP was normalized as the standard position (head-of-bed, HoB) elevation to 30°.

No significant relationship was observed between the risk of periprocedural intracranial hypertension and ICU and hospital LOS, the duration of mechanical ventilation, or the duration of mechanical ventilation after tracheostomy (Table 4). We observed a significant relationship between TIL recorded on the day of tracheostomy and the risk of raised intracranial pressure; patients in the H-ICP group more frequently presented with TIL 3–4 compared to patients in the N-ICP group (*p* = 0.005, odds ratio [OR] 5.4, confidence interval [CI] 1.5–18.7) (Table 1). SAH severity and aneurysm management, preprocedural sedation, plasma sodium concentration, and body temperature did not appear to be significantly associated with the risk of increased ICP during tracheostomy (Tables 1, 2).

**Table 4:** ICU and hospital length of stay, duration of mechanical ventilation, and days on tracheostomy stratified to normal versus high ICP status.

		Normal ICP (*n* = 30)	High ICP (*n* = 20)	*p* value
ICU LOS (days)	Median (IQR)	25 (6)	24.5 (17)	0.744
Hospital LOS (days)	Median (IQR)	38 (31)	62 (70)	0.157
Ventilation (days)	Median (IQR)	24 (8)	23 (23)	0.968
Decannulation (day)	Median (IQR)	46 (68)	66 (65)	0.887

ICP, intracranial pressure; ICU, intensive care unit; LOS, length of stay; IQR, interquartile range.

The mean duration of tracheostomy was 9 minutes (±7): 10 minutes (±8) in the H-ICP group versus 8 min (±6) in the N-ICP group (Table 2).

### Secondary endpoints

Considering the entire population, a CPP decrease below 65 mmHg occurred at least once in 32 patients (64%), but CPP variation over the five time points was not significant (Table 3; Figure 2B). However, in the H-ICP group, compared to the N-ICP group, a significantly lower CPP was observed at T3 and T4. None of the patients presented with CPP < 65 mmHg at T5; a mean CPP of 72 mmHg (±8) was recorded at this last time point. The MAP increased significantly throughout the procedure, from baseline to cannulation, and then decreased at T5 (Figure 2C).

**Figure 3:**
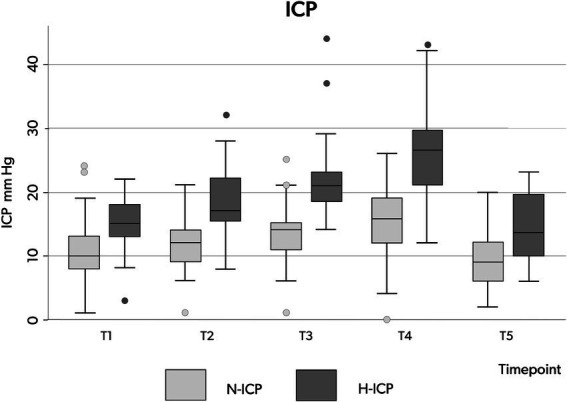
Box plots illustrating the distribution of ICP values (minimum value, first quartile, median, third quartile, and maximum value) at the five time points in patients presenting with N-ICP (light gray boxes) and H-ICP (dark gray boxes). ICP, intracranial pressure; N-ICP, normal intracranial pressure; H-ICP, high intracranial pressure.

PaCO2 appeared stable across the five time points among the whole group, with no significant changes recorded (Table 3; Figure 2D). In the H-ICP group, compared to the N-ICP group, a significantly higher PaCO_2_ was observed at T4 (*p* = 0.0045, OR 1.1, CI: 1.0–1.2), as depicted in Figure 3.

We observed periprocedural PaO_2_ consistently above 150 mmHg; this parameter varied significantly during the procedure, increasing from 166 mm Hg (±111) at T1 to 351 mmHg (±121) at T2, and 359 mm Hg (±10) at T3, reaching the highest values at T4, 366 mmHg (±115), and returning to baseline values at the end of the procedure, T5, with a mean of 189 mmHg (±139; Table 3). No episodes of arterial oxygen saturation (SaO_2_) < 92% occurred during the procedures. At the end of PDT, five patients (10% of the whole group) had a PaO_2_ < 80 mmHg; alveolar recruitment maneuvers were able to revert hypoxemia in all of these patients.

During the tracheostomy procedure, two subcutaneous false passages, one tracheal ring rupture, and three self-limiting bleedings occurred. However, accidental extubation, posterior tracheal wall punctures, pneumothorax, or severe bleeding requiring surgical treatment were not incurred. The only late complication noticed during the ICU stay was the development of peristomal signs of inflammation in three patients (6%), managed with topical treatment. No late bleeding requiring surgical revision or confirmed tracheostomy infection was reported. None of the patients developed tracheal fistula, granulomas, tracheal stenosis, or required endoluminal airway repair.

**Figure 4:**
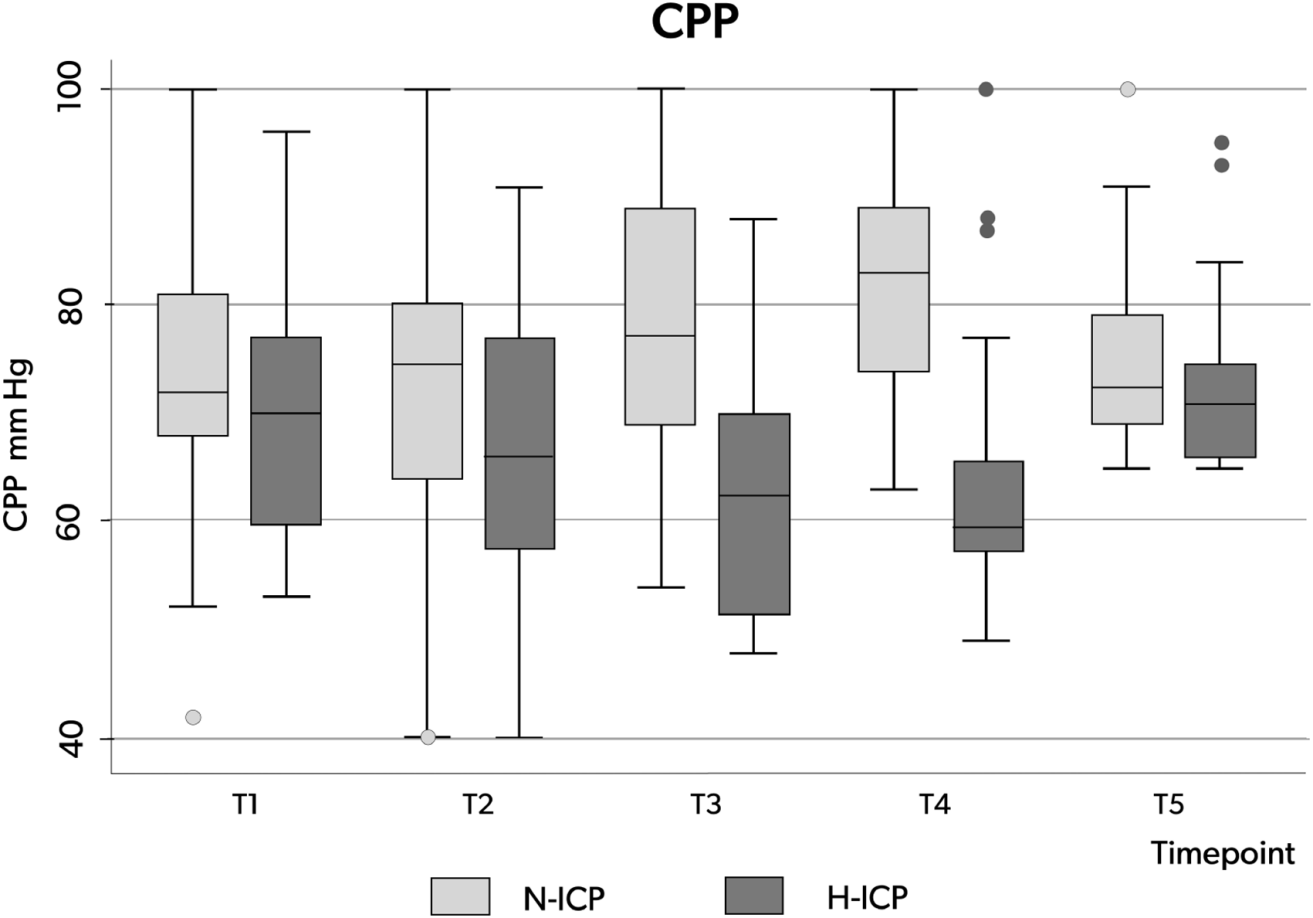
Box plots illustrating the distribution of CPP values (minimum value, first quartile, median, third quartile, and maximum value) at the five time points in patients presenting with N-ICP (light gray boxes) and H-ICP (dark boxes). CPP, cerebral perfusion pressure; N-ICP, normal intracranial pressure; H-ICP, high intracranial pressure.

**Figure 5:**
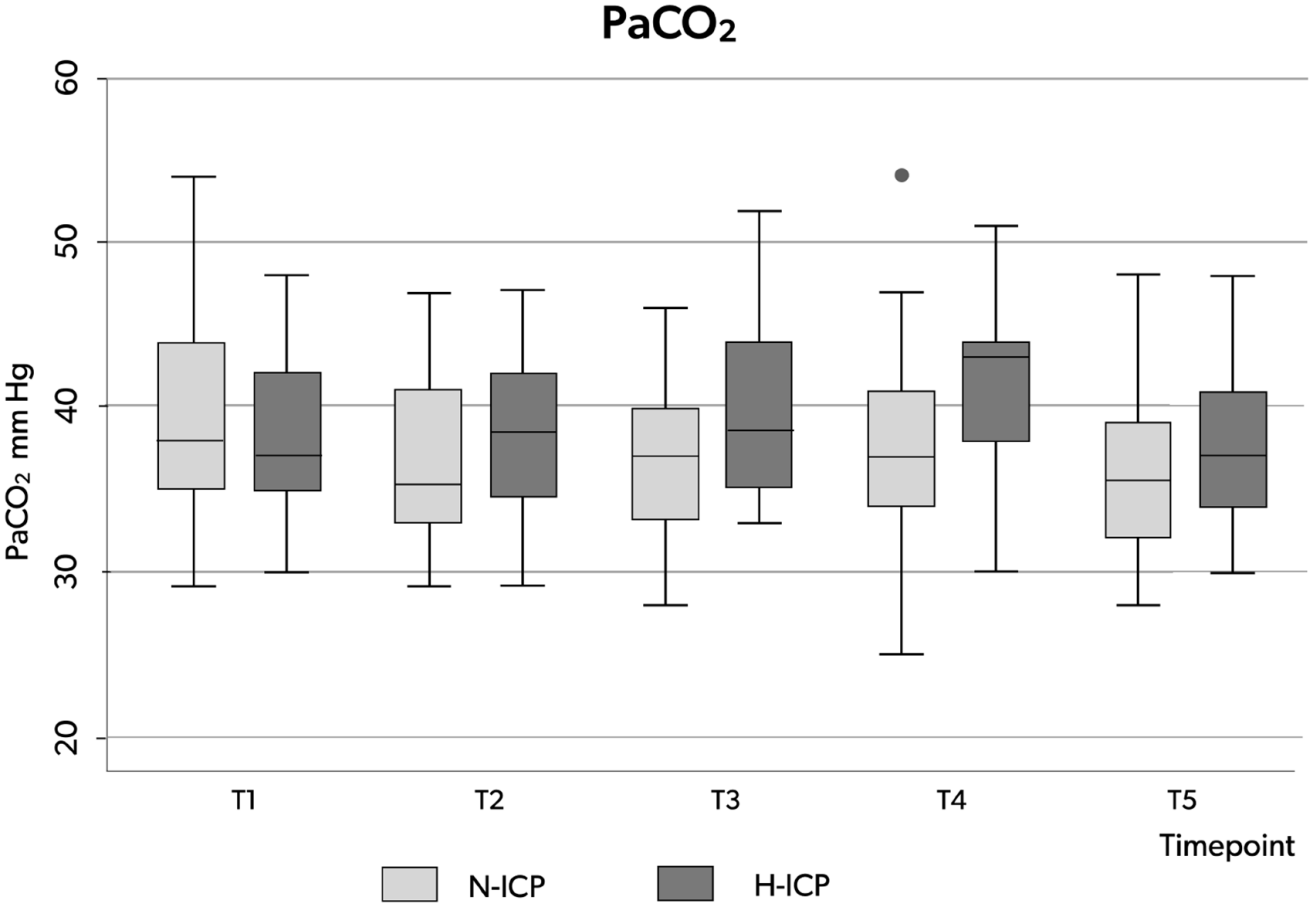
Box plots illustrating the distribution of PaCO2 values (minimum value, first quartile, median, third quartile, and maximum value) at the five time points in patients presenting with N-ICP (light gray boxes) and H-ICP (dark gray boxes). PaCO_2_, partial pressure of carbon dioxide in the arterial blood; N-ICP, normal intracranial pressure; H-ICP, high intracranial pressure.

The 6 months outcome, according to the GOS, was not significantly different between patients in the H-ICP and N-ICP groups (Table 5). At this time, 44 patients underwent decannulation. Among the 50 patients, four (8%) died in the ICU within 5–25 days after tracheostomy. One patient (2%) died at 3 months, still in the hospital post-intensive care unit, with the endotracheal cannula still in place. At the 6-month assessment, only one patient (2%) was still in the hospital; at the time of evaluation, the tracheal cannula was still in place (Figures 4, 5).

**Table 5:** GOS stratified according to normal versus high ICP status.

GOS (6 months)	Total (*n* = 50)	Normal ICP (*n* = 30)	High ICP (*n* = 20)
GR	11 (22)	5 (25)	6 (20)
MD	8 (16)	3 (15)	5 (17)
SD	22 (44)	9 (45)	13 (43)
*VS*	4 (8)	1 (5)	3 (10)
D	5 (10)	2 (10)	3 (10)

ICP, intracranial pressure; Glasgow Outcome Scale. Data reported as No. (%).

The mean duration of invasive mechanical ventilation was 31 days (±19), with a minimum of 13 days and a maximum of 106 days. The mean time between tracheostomy and decannulation was 82 days (±54 days), with a minimum of 10 days and a maximum of 210 days. Only two patients (4%) were weaned and decannulated within 15 days. We did not observe any significant correlation between baseline patient characteristics, SAH severity, aneurysm treatment strategy, TIL, development of intracranial hypertension during PDT, duration of invasive MV, or duration of MV after tracheostomy.

## Discussion

### Intracranial pressure

In our single-center analysis, including a homogeneous population of consecutive patients with SAH admitted to the ICU after aneurysm coiling or clipping and undergoing early percutaneous dilatational tracheostomy, we observed an intraprocedural increase in intracranial pressure above 20 mm Hg in at least one of the five time points in 27 patients (54%). Twenty (40%) patients required treatment. During tracheostomy, forced hyperextended neck posture, as well as possible hypoventilation, hypercapnia, and hypertension might activate cerebral autoregulation mechanisms, resulting in swings in ICP and, eventually, CPP.

Moreover, since all patients with EVD, prior to PDT underwent continuous CSF drainage (49, 50), the temporary EVD clamp during PDT could be one of the triggers for increased ICP (57, 58).

Given these risks, periprocedural continuous monitoring of both ICP and CPP is mandatory to immediately detect abnormalities, provide corrective measures, and improve patient safety. The ICP swings appeared transitory, with the recovery of values below 20 mm Hg at the end of the procedure. The short-term duration of ICP increases, and the return to baseline is consistently described in the published literature.

Early bedside tracheostomy in an SAH is not without risks, and increased ICP during PDT was previously reported in different populations of neurocritically ill patients, including those with traumatic brain injury, ischemic stroke, brain tumors, subarachnoid hemorrhage, and intraparenchymal hemorrhage (13, 14, 33–38, 59).

Consistent with our observations, the phase of the procedure associated with the highest risk for intracranial hypertension was the insertion of the tracheal cannula (33, 36, 37).

Stocchetti et al. (36) reported a statistically significant increase in ICP above 20 mm Hg, albeit not clinically impacting, during the cannulation phase in five patients (25%) among a population of 20 patients with traumatic brain injury or cerebrovascular disease. Another group (32) reported a transient increase in ICP in 11 (17%) among a cohort of 65 neurocritically ill patients undergoing tracheostomy performed using the Percu-Twist technique (60).

Kocaeli et al. (33) compared early versus late tracheostomies performed according to the Griggs method (61) in 30 patients with TBI, ICH, SAH, or cerebral tumors. The 15 patients who underwent early tracheostomy (performed after a mean of 4.5 ± 0.6 days after ICU admission) observed an increase in ICP values up to 28.4 mm Hg (±13.7) during tracheal cannula insertion, with no significant difference between early and late tracheostomies. The ICP value is higher than the mean 19.1 mm Hg (±8.5 mmHg) we observed. In a consistent proportion of our patients (46 patients, 92% of the whole population), the presence of a ventricular catheter, allowing for eventual deliquoration, could have contributed to the maintenance of an ICP below 20 mm Hg. Liquor drainage was the strategy we and other groups (27) most frequently used to manage intraprocedural HICP. Additional sedation and osmotic therapy were used in seven (14%) and five patients (10%), respectively. The same approach has been described previously (13, 32, 59).

In recent years, Kuechler et al. (37) published a study that included data on 289 patients with TBI, SAH, ICH, or brain tumors. They observed a transient ICP increase above 20 mmHg in 98 patients (34%) during tracheostomy. In a heterogeneous population of neuro-intensive care patients, temporary increases in ICP, occurring at any time during the tracheostomy procedure and eventually requiring treatment, were detected in a variable percentage of patients (38–65%) (13, 59).

We confirmed, as described by Kleffmann et al., that the presence of higher ICP at baseline represents a risk factor for the development of HICP (59). Moreover, the correlation between the occurrence of HICP and higher TIL appears significant. It is likely that the need for higher-level therapies to maintain a normal ICP on the day of tracheostomy is an indirect sign of reduced cerebral compliance and, thus, an increased risk of HICP in the case of forced posture for surgery or hypercapnia from relative hypoventilation.

We did not observe any relationship between temporary ICP increases during tracheostomy and baseline factors, disease severity, or treatment by coiling or clipping. A direct comparison with other case studies is not feasible because a selected population of patients with SAH has not been investigated by other authors.

In our experience including patients with SAH, transient intraprocedural endocranial hypertension did not correlate with worst prognosis. Indeed, the clinical outcome of both H-ICP and N-ICP groups is consistent.

Our results refer to an extremely selected population and, therefore, cannot be generalized to all neurocritically ill patients.

### Cerebral pressure perfusion and PaCO_2_

A decrease in CPP during the tracheostomy procedure has been reported (13, 59), but other authors did not notice a significant change in CPP (37). In our patients, vasopressors increased MAP consensually with the eventual increase in ICP during airway manipulation. This could have contributed to the maintenance of CPP consistently above 65 mmHg at all time points.

Increased PaCO_2_ is considered a major determinant of HICP by several authors (13, 37, 47, 48, 59).

The presence of a fiber-optic endoscope and the switch from the orotracheal tube to the tracheal cannula might decrease tidal volume, with possible hypercapnia, which, activating cerebral metabolic autoregulation, might induce cerebral vasodilation and, thus, transient intracranial hypertension. Kuechler et al. (37) reported a relationship between variations in PaCO_2_ and ICP, with hypercapnia appearing in one-third of the patients. Despite meticulous management of airways and MV during tracheostomy, we observed a statistically significantly higher PaCO_2_ during the cannulation phase among patients in the H-ICP group than in the N-ICP group.

Transient hypercapnia is likely the main cause of intracranial hypertension during the procedure because of the partially occluding effect of the bronchoscope (T3) and the passage from the tracheal tube to the cannula (T4). The decision to perform tracheostomy in patients with SAH requires careful evaluation of the potential associated advantages and risks of procedure-induced intracranial hypertension.

### Timing of tracheostomy

The optimal timing of tracheostomy in the general population of critically ill patients is still controversial; many articles do not show any advantage in the use of early tracheostomy (14, 62, 63); other authors emphasized the potential advantage in decreased need for sedation, fewer days on mechanical ventilation and hospitalization, and, eventually, improved outcomes (54, 55).

Even with referral to neurocritical patients no definitive recommendations exists supporting the standardization of timing for tracheostomy.

The SETPOINT and SETPOINT II prospective studies, including patients with AIS, ICH, and SAH (16, 64) show no statistically significant benefits on mortality or neurological outcome. This is consistent with the ESICM Recommendations on ventilation in patients with acute brain injury (2).

Many authors, however, suggested that early tracheostomy might increase patients’ comfort, potentially facilitating weaning from sedation, improve neurological evaluation, decrease the duration of MV and the risk of ventilator-associated events (VAEs), and reduce the ICU length of stay (LoS) and the associated costs (6, 15–18, 64–66) in neurocritical patients.

In the few studies focusing only on SAH (5, 24, 58), no benefit on mortality was demonstrated with early tracheostomy, but patients who underwent early tracheostomy had fewer episodes of pneumonia, spent fewer days on mechanical ventilation, and were decannulated earlier (5). The fact that no patient was decannulated before 14 days could suggest the low incidence of unneeded tracheostomy. Colombo et al. (58) also reported a lower incidence of pneumonia, a shorter duration of MV and ICU LoS in patients with SAH.

Predicting outcome in poor-grade SAH is challenging, as defining the optimal timing of tracheostomy. Since an eventual transient increase in ICP does not worsen the outcome, and considering the reduction in infectious complications and days on ventilator, early tracheotomy could be a good option, from risk/benefit ratio perspective (24).

Daily evaluation of TIL, ICP values over the previous 24 h, and a pre-procedure DVE closure test can help in identifying the optimal timing for tracheostomy. The pathophysiology of SAH, characterized by an early brain insult and a possible secondary hit due to vasospasm, and inducing late cerebral ischemia, leads to the hypothesis of finding an appropriate window of opportunity to safely perform tracheotomy between the third and fifth day since the event.

### Complications

According to a 2013 meta-analysis (26), including 24,307 patients, the rate of fatal complications was extremely low (0.17%). None of the patients died as a consequence of the procedure.

Immediate complications are described in 3–6% of procedures (25, 26, 42, 59, 67, 68).

We observed minor bleeding in 3 patients (6%); a variable ratio of 1.6 to 14% of minor bleeding has been reported (59, 68) major bleeding did not occur in our experience, and some authors reported a complication occurring in 0.5–1.5% of PDTs (32, 37, 42, 68).

Previous data reported the need to convert from PDT to surgical tracheostomy in 1–7.7% of procedures (25, 32), and no need to interrupt the procedure.

We observed two episodes of false passage and 2% of tracheal ring ruptures, previously reported as occurring in about 1% (42, 68) and 5–36% of procedures, respectively, with inconsistent definitions and unclear relationship with the development of late tracheal stenosis (68).

Signs of local inflammation were observed in a small proportion of patients (6%) without signs of cellulitis. Moreover, we did not observe major late complications (tracheal stenosis, granulomas, or fistulas requiring surgical treatment). The incidence of these complications in published experience has reached up to 4% (42, 68); specifically, an incidence of up to 1.6% of tracheal stenosis (42, 68), and up to 4% of severe infections with cellulitis have been previously reported (21, 42).

The low incidence of complications we reported is likely due to a well-defined strategy for PDT coupled with a standardized management protocol implemented by a limited pool of experienced physicians and nurses.

### Limitations and strengths

To the best of our knowledge, this is the first study to investigate the trends in ICP and CPP values during PDT in a homogeneous cohort of patients with SAH. The monocentric design allowed for a high level of standardization of tracheostomy procedures, managed by a dedicated “tracheo-team” implementing a strictly defined protocol if intracranial hypertension was detected. The same team strictly monitored the patients post-procedure and implemented accurate follow-up to detect early and late complications, avoiding potential underestimation. The limited size, and the selected population, prevents generalizability of results, which could be considered as hypothesis generating.

We recorded ICP and CPP at five predefined crucial time points; this non-continuous monitoring could have overestimated or underestimated their real trend. Continuous data collection would have made it possible to accurately measure ICP dose, relevant factors affecting secondary brain injury, and patient outcomes (3, 69).

A comparison between early and late PDT was not possible as we considered early tracheostomy as the first choice in SAH at the time of designing the study.

External ventricular drainage was required in most of our patients due to the presence of intraventricular hemorrhage and the high risk of developing hydrocephalus; therefore, no comparison was feasible with patients presenting with intraparenchymal rather than ventricular catheters.

Detecting cerebral arterial vasospasm was beyond the study’s aims; moreover, procedures were mostly performed before the time considered at the highest risk of this complication.

## Conclusion

We conducted a single-center, prospective, observational study including a homogeneous sample of consecutive adult patients with subarachnoid hemorrhage admitted to the ICU and undergoing aneurysm securing through coiling or clipping who required early tracheostomy and received ICP monitoring during the procedure. This study aimed to assess PDT-associated variations in intracranial pressure (ICP), cerebral perfusion pressure (CPP), and other main hemodynamic and respiratory variables, the occurrence of tracheostomy-related complications, and their relationship with outcomes in a selected cohort.

We observed a temporary increase in intracranial pressure, reduction in CPP, and increase in PaCO2 without reduction of PaO2 during early percutaneous dilatational tracheostomy. These brief episodes of intracranial hypertension appeared mainly due to the activation of cerebral autoregulatory mechanisms in patients with impaired compensatory mechanisms and compliance, without supporting evidence of impact on short and long-term outcome.

The low number of observed complications might be related to our organizational strategy, all based on a dedicated “tracheo-team” implementing both PDT following a strictly defined protocol and accurate follow-up.

Further prospective studies, including a larger number of patients with SAH and comparing different management strategies, are needed to better define tracheostomy timing and approach in this cohort.

## Data availability statement

The raw data supporting the conclusions of this article will be made available by the authors, without undue reservation.

## Ethics statement

The studies involving human participants were reviewed and approved by CEROM Comitato Etico della Romagna. The ethics committee waived the requirement of written informed consent for participation.

## Author contributions

GB: study design and manuscript writing. ER and MA: data analysis and manuscript review. EP, VB, GP, AR, and FZ: data collection. VA: manuscript review. All authors contributed to the article and approved the submitted version.

## Conflict of interest

The authors declare that the research was conducted in the absence of any commercial or financial relationships that could be construed as a potential conflict of interest.

## Publisher’s note

All claims expressed in this article are solely those of the authors and do not necessarily represent those of their affiliated organizations, or those of the publisher, the editors and the reviewers. Any product that may be evaluated in this article, or claim that may be made by its manufacturer, is not guaranteed or endorsed by the publisher.

## References

[1] Zoerle T Lombardo A Colombo A Longhi L Zanier ER Rampini P. Intracranial pressure after subarachnoid hemorrhage. Crit Care Med. (2015) 43:168–76. doi: 10.1097/CCM.0000000000000670, PMID: 25318385

[2] Chang YM Lee TH Liao CC Huang YH. Characterization of tracheotomized patients after spontaneous subarachnoid hemorrhage. Medicine (Baltimore). (2020) 99:e21057. doi: 10.1097/MD.0000000000021057, PMID: 32664119 PMC7360272

[3] Carra G Elli F Ianosi B Flechet M Huber L Rass V. Association of Dose of intracranial hypertension with outcome in subarachnoid hemorrhage. Neurocrit Care. (2021) 34:722–30. doi: 10.1007/s12028-021-01221-4, PMID: 33846900

[4] Ryttlefors M Howells T Nilsson P Ronne-Engström E Enblad P. Secondary insults in subarachnoid hemorrhage: occurrence and impact on outcome and clinical deterioration. Neurosurgery. (2007) 61:704–15. doi: 10.1227/01.NEU.0000298898.38979.E3, PMID: 17986931

[5] Gessler F Mutlak H Lamb S Hartwich M Adelmann M Platz J. The impact of tracheostomy timing on clinical outcome and adverse events in poor-grade subarachnoid hemorrhage. Crit Care Med. (2015) 43:2429–38. doi: 10.1097/CCM.0000000000001195, PMID: 26308429

[6] Dasenbrock HH Rudy RF Gormley WB Frerichs KU Aziz-Sultan MA Du R. The timing of tracheostomy and outcomes after aneurysmal subarachnoid hemorrhage: a Nationwide inpatient sample analysis. Neurocrit Care. (2018) 29:326–35. doi: 10.1007/s12028-018-0619-4, PMID: 30298335

[7] Magni F Pozzi M Rota M Vargiolu A Citerio G. High-resolution intracranial pressure burden and outcome in subarachnoid hemorrhage. Stroke. (2015) 46:2464–9. doi: 10.1161/STROKEAHA.115.010219, PMID: 26243224

[8] Report of world Federation of Neurological Surgeons Committee on a universal subarachnoid hemorrhage grading scale. J Neurosurg. (1988) 68:985–6. doi: 10.3171/jns.1988.68.6.0985, PMID: 3131498

[9] Hunt WE Hess RM. Surgical risk as related to time of intervention in the repair of intracranial aneurysms. J Neurosurg. (1968) 28:14–20. doi: 10.3171/jns.1968.28.1.0014, PMID: 5635959

[10] Cinotti R Mijangos JC Pelosi P Haenggi M Gurjar M Schultz MJ. Extubation in neurocritical care patients: the ENIO international prospective study. Intensive Care Med. (2022) 48:1539–50. doi: 10.1007/s00134-022-06825-8 36038713

[11] Godet T Chabanne R Marin J Kauffmann S Futier E Pereira B. Extubation failure in brain-injured patients: risk factors and development of a prediction score in a preliminary prospective cohort study. Anesthesiology. (2017) 126:104–14. doi: 10.1097/ALN.0000000000001379, PMID: 27749290

[12] Asehnoune K Seguin P Lasocki S Roquilly A Delater A Gros A. ATLANREA group. Extubation success prediction in a multicentric cohort of patients with severe brain injury. Anesthesiology. (2017) 127:338–46. doi: 10.1097/ALN.0000000000001725, PMID: 28640020

[13] Kleffmann J Pahl R Deinsberger W Ferbert A Roth C. Effect of percutaneous tracheostomy on intracerebral pressure and perfusion pressure in patients with acute cerebral dysfunction (TIP trial): an observational study. Neurocrit Care. (2012) 17:85–9. doi: 10.1007/s12028-012-9709-x, PMID: 22539153

[14] Andriolo BN Andriolo RB Saconato H Atallah ÁN Valente O Cochrane Emergency and Critical Care Group. Early versus late tracheostomy for critically ill patients. Cochrane Database Syst Rev. (2015) 2018:CD007271. doi: 10.1002/14651858.CD007271.pub3, PMID: 25581416 PMC6517297

[15] Robba C Poole D McNett M Asehnoune K Bösel J Bruder N. Mechanical ventilation in patients with acute brain injury: recommendations of the European Society of Intensive Care Medicine consensus. Intensive Care Med. (2020) 46:2397–410. doi: 10.1007/s00134-020-06283-0, PMID: 33175276 PMC7655906

[16] Bösel J Schiller P Hook Y Andes M Neumann JO Poli S. Stroke-related early tracheostomy versus prolonged Orotracheal intubation in Neurocritical care trial (SETPOINT): a randomized pilot trial. Stroke. (2013) 44:21–8. doi: 10.1161/STROKEAHA.112.669895, PMID: 23204058

[17] McCredie VA Alali AS Scales DC Adhikari NK Rubenfeld GD Cuthbertson BH. Effect of early versus late tracheostomy or prolonged intubation in critically ill patients with acute brain injury: a systematic review and meta-analysis. Neurocrit Care. (2017) 26:14–25. doi: 10.1007/s12028-016-0297-z, PMID: 27601069

[18] Rizk EB Patel AS Stetter CM Chinchilli VM Cockroft KM. Impact of tracheostomy timing on outcome after severe head injury. Neurocrit Care. (2011) 15:481–9. doi: 10.1007/s12028-011-9615-7, PMID: 21786043

[19] Villwock JA Villwock MR Deshaies EM. Tracheostomy timing affects stroke recovery. J Stroke Cerebrovasc Dis. (2014) 23:1069–72. doi: 10.1016/j.jstrokecerebrovasdis.2013.09.008, PMID: 24555919

[20] Alsherbini K Goyal N Metter EJ Pandhi A Tsivgoulis G Huffstatler T. Predictors for tracheostomy with external validation of the stroke-related early tracheostomy score (SETscore). Neurocrit Care. (2019) 30:185–92. doi: 10.1007/s12028-018-0596-7, PMID: 30167898

[21] Siddiqui UT Tahir MZ Shamim MS Enam SA. Clinical outcome and cost effectiveness of early tracheostomy in isolated severe head injury patients. Surg Neurol Int. (2015) 6:65. doi: 10.4103/2152-7806.155757, PMID: 25984381 PMC4418102

[22] Chen W Liu F Chen J Ma L Li G You C. Timing and outcomes of tracheostomy in patients with hemorrhagic stroke. World Neurosurg. (2019) 131:e606–13. doi: 10.1016/j.wneu.2019.08.013, PMID: 31408751

[23] Hyde GA Savage SA Zarzaur BL Hart-Hyde JE Schaefer CB Croce MA. Early tracheostomy in trauma patients saves time and money. Injury. (2015) 46:110–4. doi: 10.1016/j.injury.2014.08.049, PMID: 25441577

[24] Wolf S. Tracheostomy in poor-grade subarachnoid hemorrhage: if deemed necessary, You may want to perform it early. Crit Care Med. (2015) 43:2514–5. doi: 10.1097/CCM.0000000000001245, PMID: 26468707

[25] Higgins KM Punthakee X. Meta-analysis comparison of open versus percutaneous tracheostomy. Laryngoscope. (2007) 117:447–54. doi: 10.1097/01.mlg.0000251585.31778.c9, PMID: 17334304

[26] Simon M Metschke M Braune SA Püschel K Kluge S. Death after percutaneous dilatational tracheostomy: a systematic review and analysis of risk factors. Crit Care. (2013) 17:R258. doi: 10.1186/cc13085, PMID: 24168826 PMC4056379

[27] Delaney A Bagshaw SM Nalos M. Percutaneous dilatational tracheostomy versus surgical tracheostomy in critically ill patients: a systematic review and meta-analysis. Crit Care. (2006) 10:R55. doi: 10.1186/cc4887, PMID: 16606435 PMC1550905

[28] Freeman BD Morris PE. Tracheostomy practice in adults with acute respiratory failure. Crit Care Med. (2012) 40:2890–6. doi: 10.1097/CCM.0b013e31825bc948, PMID: 22824938

[29] Durbin CG. Techniques for performing tracheostomy. Respir Care. (2005) 50:488–96. 15807911

[30] Putensen C Theuerkauf N Guenther U Vargas M Pelosi P. Percutaneous and surgical tracheostomy in critically ill adult patients: a meta-analysis. Crit Care. (2014) 18:544. doi: 10.1186/s13054-014-0544-7, PMID: 25526983 PMC4293819

[31] Seder DB Lee K Rahman C Rossan-Raghunath N Fernandez L Rincon F. Safety and feasibility of percutaneous tracheostomy performed by neurointensivists. Neurocrit Care. (2009) 10:264–8. doi: 10.1007/s12028-008-9174-8, PMID: 19130311

[32] Imperiale C Magni G Favaro R Rosa G. Intracranial pressure monitoring during percutaneous tracheostomy "percutwist" in critically ill neurosurgery patients. Anesth Analg. (2009) 108:588–92. doi: 10.1213/ane.0b013e31818f601b, PMID: 19151293

[33] Kocaeli H Korfali E Taşkapilioğlu O Ozcan T. Analysis of intracranial pressure changes during early versus late percutaneous tracheostomy in a neuro-intensive care unit. Acta Neurochir. (2008 Dec) 150:1263–7; discussion 1267. doi: 10.1007/s00701-008-0153-9, PMID: 19002373

[34] Rabinstein AA Carhuapoma JR Derdeyn CP Dion J Higashida RT Hoh BL. Guidelines for the management of aneurysmal subarachnoid hemorrhage: a guideline for healthcare professionals from the American Heart Association/american Stroke Association. Stroke. (2012) 43:1711–37. doi: 10.1161/STR.0b013e3182587839, PMID: 22556195

[35] Baggiani M Graziano F Rebora P Robba C Guglielmi A Galimberti S. Intracranial pressure monitoring practice, treatment, and effect on outcome in aneurysmal subarachnoid hemorrhage. Neurocrit Care. (2022). doi: 10.1007/s12028-022-01651-8 [Epub ahead of print]., PMID: 36471182

[36] Stocchetti N Parma A Songa V Colombo A Lamperti M Tognini L. Early translaryngeal tracheostomy in patients with severe brain damage. Intensive Care Med. (2000) 26:1101–7. doi: 10.1007/s001340051324, PMID: 11030167

[37] Kuechler JN Abusamha A Ziemann S Tronnier VM Gliemroth J. Impact of percutaneous dilatational tracheostomy in brain injured patients. Clin Neurol Neurosurg. (2015) 137:137–41. doi: 10.1016/j.clineuro.2015.07.007, PMID: 26189073

[38] Milanchi S Magner D Wilson MT Mirocha J Margulies DR. Percutaneous tracheostomy in neurosurgical patients with intracranial pressure monitoring is safe. J Trauma. (2008) 65:73–9. doi: 10.1097/TA.0b013e31814693f2, PMID: 18580518

[39] World Medical Association. World medical association declaration of Helsinki: ethical principles for medical research involving human subjects. JAMA. (2013) 310:2191–4. doi: 10.1001/jama.2013.281053, PMID: 24141714

[40] Fisher CM Kistler JP Davis JM. Relation of cerebral vasospasm to subarachnoid hemorrhage visualized by computerized tomographic scanning. Neurosurgery. (1980) 6:1–9. doi: 10.1227/00006123-198001000-00001, PMID: 7354892

[41] Maas AI Harrison-Felix CL Menon D Adelson PD Balkin T Bullock R. Standardizing data collection in traumatic brain injury. J Neurotrauma. (2011) 28:177–87. doi: 10.1089/neu.2010.1617, PMID: 21162610 PMC3037806

[42] Fikkers BG Briedé IS Verwiel JM Van Den Hoogen FJ. Percutaneous tracheostomy with the blue rhino trade mark technique: presentation of 100 consecutive patients. Anaesthesia. (2002) 57:1094–7. doi: 10.1046/j.1365-2044.2002.02834.x, PMID: 12392457

[43] Portolani L Pirini E Rasi A Ceccarelli P Dradi U Ravaldini M. Il tracheo-team nella gestione della pressione endocranica durante tracheostomia dilatativa nel trauma cranico grave: l’impatto di una checklist [the tracheo-team in the management of intracranic pressure during a dilatative tracheostomy in severe head trauma: the impact of a checklist]. Assist Inferm Ric. (2018) 37:189–95. doi: 10.1702/3080.30723 30638203

[44] Ciaglia P Firsching R Syniec C. Elective percutaneous dilatational tracheostomy. A new simple bedside procedure; preliminary report. Chest. (1985) 87:715–9. doi: 10.1378/chest.87.6.715, PMID: 3996056

[45] Byhahn C Lischke V Halbig S Scheifler G Westphal K. Ciaglia blue rhino: Ein weiterentwickeltes Verfahren der perkutanen Dilatationstracheotomie. Technik und erste klinische Ergebnisse [Ciaglia blue rhino: a modified technique for percutaneous dilatation tracheostomy. Technique and early clinical results]. Anaesthesist. (2000) 49:202–26. doi: 10.1007/s001010050815 10788989

[46] Hinerman R Alvarez F Keller CA. Outcome of bedside percutaneous tracheostomy with bronchoscopic guidance. Intensive Care Med. (2000) 26:1850–6. doi: 10.1007/s001340000718, PMID: 11271095

[47] Ghattas C Alsunaid S Pickering EM Holden VK. State of the art: percutaneous tracheostomy in the intensive care unit. J Thorac Dis. (2021) 13:5261–76. doi: 10.21037/jtd-19-4121, PMID: 34527365 PMC8411160

[48] Karagiannidis C Merten ML Heunks L Strassmann SE Schäfer S Magnet F. Respiratory acidosis during bronchoscopy-guided percutaneous dilatational tracheostomy: impact of ventilator settings and endotracheal tube size. BMC Anesthesiol. (2019) 19:147. doi: 10.1186/s12871-019-0824-5, PMID: 31399057 PMC6689167

[49] Chung DY Leslie-Mazwi TM Patel AB Rordorf GA. Management of External Ventricular Drains after subarachnoid hemorrhage: a multi-istitutional survey. Neurocrit Care. (2017) 26:356–61. doi: 10.1007/s12028-016-0352-9, PMID: 28000129 PMC5444979

[50] Qian C Yu X Chen J Gu C Wang L Chen G. Effect of the drainage of cerebrospinal fluid in patients with aneurismal subaracnoid hemorrhage. A meta-analysis. Medicine (Baltimore). (2016) 95:e5140. doi: 10.1097/MD.0000000000005140, PMID: 27741143 PMC5072970

[51] Ravishankar N Nuoman R Amuluru K El-Ghanem M Thulasi V Dangayach NS. Management strategies for intracranial pressure crises in subarachnoid hemorrhage. J Intensive Care Med. (2020) 35:211–8. doi: 10.1177/0885066618813073, PMID: 30514150

[52] Jennett B Bond M. Assessment of outcome after severe brain damage. Lancet. (1975) 305:480–4. doi: 10.1016/S0140-6736(75)92830-5, PMID: 46957

[53] von Elm E Altman DG Egger M Pocock SJ Gøtzsche PC Vandenbroucke PC. The Strengthening the Reporting of Observational Studies in Epidemiology (STROBE) Statement: guidelines for reporting observational studies. Int J Surg. (2014) 12:1495–9. doi: 10.1016/j.ijsu.2014.07.013 25046131

[54] Shemie SD Hornby L Baker A Teitelbaum J Torrance S Young K. International guideline development for the determination of death. Intensive Care Med. (2014) 40:788–97. doi: 10.1007/s00134-014-3242-7, PMID: 24664151 PMC4028548

[55] Manara AR Thomas I Harding R. A case for stopping the early withdrawal of life sustaining therapies in patients with devastating brain injuries. J Intensive Care Soc. (2016) 17:295–301. doi: 10.1177/1751143716647980, PMID: 28979514 PMC5624473

[56] Harvey D Butler J Groves J Manara A Menon D Thomas E. Management of perceived devastating brain injury after hospital admission: a consensus statement from stakeholder professional organizations. Br J Anaesth. (2018) 120:138–45. doi: 10.1016/j.bja.2017.10.002, PMID: 29397121

[57] Chaikittisilpa N Lele AV Lyons VH Nair BG Newman S-F Blissitt PA. Risks of routinely clamping external ventricular drains dor intrahospital transport in neurocritically ill cerebrovascular patients. Neurocrit Care. (2017) 26:196–204. doi: 10.1007/s12028-016-0308-0, PMID: 27757914

[58] Colombo J Perego M Veronese G Zumbo F Pressato L Curto F. The RAISE score: a possible tool to better identify subarachnoid hemorrhage patients who might benefit from early tracheostomy? Crit Care Med. (2022) 50:e329–30. doi: 10.1097/CCM.0000000000005367, PMID: 35191885

[59] Kleffmann J Pahl R Ferbert A Roth C. Factors influencing intracranial pressure (ICP) during percutaneous tracheostomy. Clin Neurol Neurosurg. (2020) 195:105869. doi: 10.1016/j.clineuro.2020.105869, PMID: 32353664

[60] Westphal K Maeser D Scheifler G Lischke V Byhahn C. PercuTwist: a new single-dilator technique for percutaneous tracheostomy. Anesth Analg. (2003) 96:229–32. doi: 10.1097/00000539-200301000-00046 12505957

[61] Griggs WM Worthley LI Gilligan JE Thomas PD Myburg JA. A simple percutaneous tracheostomy technique. Surg Gynecol Obstet. (1990) 170:543–5. 2343371

[62] Terragni PP Antonelli M Fumagalli R Faggiano C Berardino M Pallavicini FB. Early vs late tracheotomy for prevention of pneumonia in mechanically ventilated adult ICU patients: a randomized controlled trial. JAMA. (2010) 303:1483–9. doi: 10.1001/jama.2010.447, PMID: 20407057

[63] Young D Harrison DA Cuthbertson BH Rowan K Collaborators TM. Effect of early vs late tracheostomy placement on survival in patients receiving mechanical ventilation: the TracMan randomized trial. JAMA. (2013) 309:2121–9. doi: 10.1001/jama.2013.5154, PMID: 23695482

[64] Bösel J Niesen WD Salih F. Effect of early vs standard approach to tracheostomy on functional outcome at 6 months among patients with severe stroke receiving mechanical ventilation: the SETPOINT2 randomized clinical trial. JAMA. (2022) 327:1899–909. doi: 10.1001/jama.2022.4798, PMID: 35506515 PMC9069344

[65] Hosokawa K Nishimura M Egi M Vincent JL. Timing of tracheotomy in ICU patients: a systematic review of randomized controlled trials. Crit Care. (2015) 19:424. doi: 10.1186/s13054-015-1138-8, PMID: 26635016 PMC4669624

[66] Tanaka A Uchiyama A Kitamura T Sakaguchi R Komukai S Matsuyama T. Association between early tracheostomy and patient outcomes in critically ill patients on mechanical ventilation: a multicenter cohort study. J Intensive Care. (2022) 10:19. doi: 10.1186/s40560-022-00610-x, PMID: 35410403 PMC8996211

[67] Bösel J. Use and timing of tracheostomy after severe stroke. Stroke. (2017) 48:2638–43. doi: 10.1161/STROKEAHA.117.017794, PMID: 28733479

[68] Dempsey GA Grant CA Jones TM. Percutaneous tracheostomy: a 6 yr prospective evaluation of the single tapered dilator technique. Br J Anaesth. (2010) 105:782–8. doi: 10.1093/bja/aeq238, PMID: 20813838

[69] Güiza F Depreitere B Piper I Citerio G Chambers I Jones PA. Visualizing the pressure and time burden of intracranial hypertension in adult and paediatric traumatic brain injury. Intensive Care Med. (2015) 41:1067–76. doi: 10.1007/s00134-015-3806-1, PMID: 25894624

